# Effect of Dietary Berry Supplementation on Antioxidant Biomarkers in Adults with Cardiometabolic Risks: A Systematic Review of Clinical Trials

**DOI:** 10.3390/antiox12061182

**Published:** 2023-05-30

**Authors:** Macy M. Helm, Tolu Alaba, Dorothy Klimis-Zacas, Kenneth Izuora, Arpita Basu

**Affiliations:** 1 Department of Kinesiology and Nutrition Sciences, School of Integrated Health Sciences, University of Nevada, Las Vegas, NV 89154, USA; helmm1@unlv.nevada.edu; 2 School of Food and Agriculture, University of Maine, Orono, ME 04469, USA; tolu.adekeye@maine.edu (T.A.); dorothea@maine.edu (D.K.-Z.); 3 Graduate School of Biomedical Sciences and Engineering, University of Maine, Orono, ME 04469, USA; 4 Section of Endocrinology, Department of Internal Medicine, University of Nevada, Las Vegas, NV 89102, USA; kenneth.izuora@unlv.edu

**Keywords:** oxidative stress, cardiovascular, metabolic syndrome, overweight, obesity, strawberries, blueberries, cranberries

## Abstract

Cardiometabolic conditions are closely associated with inflammation and oxidative stress. Dietary berries may serve as a beneficial nutrition intervention to address the features of cardiometabolic dysfunction and associated oxidative stress. The high antioxidant status of dietary berries may increase antioxidant capacity and reduce biomarkers of oxidative stress. This systematic review was conducted to investigate these effects of dietary berries. The search was conducted using PubMed, Cochrane Library, Web of Science, and citation searching. Through this search we identified 6309 articles and 54 were included in the review. Each study’s risk of bias was assessed using the 2019 Cochrane Methods’ Risk of Bias 2 tool. Antioxidant and oxidative stress outcomes were evaluated, and the magnitude of effect was calculated using Cohen’s *d*. A range of effectiveness was reported in the included studies and the quality of the studies differed between the parallel and crossover trials. Considering the inconsistency in reported effectiveness, future investigations are warranted to determine the acute and sustained reductions of oxidative stress biomarkers from dietary berry intake (PROSPERO registration# CRD42022374654).

## 1. Introduction

Cardiometabolic diseases represent four of the ten leading causes of death in the United States [1]. Between 1990 and 2017, cardiometabolic diseases accounted for nearly 5 million deaths of working-age adults [2]. A cluster of pathologies typify cardiovascular and metabolic diseases, including hypertension, insulin resistance, dyslipidemia, and visceral adiposity [3]. This cluster of pathologies is associated with inflammation due to the causality between active immune system and metabolic impairments [4]. Cardiometabolic risk factors can promote inflammation and be an outcome of exacerbated inflammatory processes. Hypertension increases the circulation of cytotoxic T cells, creating a pro-inflammatory physiological state [5]. Visceral adiposity is also a pro-inflammatory state that stimulates the immune system production of cytokines [6]. Inflammation disrupts insulin action and secretion [4], potentially contributing to insulin resistance. Similarly, cytokine inflammatory markers contribute to the accumulation of cholesterol seen in dyslipidemia [7].

Oxidative stress plays a major role in the pathology of inflammation and associated cardiometabolic diseases. While it is a complex cascade of physiological processes, oxidative stress can simply be explained as an imbalance of reactive oxygen species and antioxidants [8]. This imbalance is associated with the pathogenesis of cardiometabolic diseases and thus is a target for risk factor management [8]. Dietary approaches have emerged as one method of addressing the risk factors of cardiometabolic conditions, potentially by modulating oxidative stress. Antioxidant status can be measured using endogenous antioxidant enzymes, such as catalase and the glutathione antioxidant system, as well as exogenous antioxidants in the form of serum vitamins C and E [9,10,11].

Antioxidant-rich foods include those from plant sources, such as berries, fruits, vegetables, grains, and herbs. Among the commonly consumed plant foods, berries have received much attention because of their high antioxidant activity based on various types of phytochemicals including flavonoids [12]. Among the commonly consumed dietary berries, blueberries, cranberries, and strawberries have a high total polyphenol content, as well as vitamin C and E, and all of these contribute to their potent antioxidant effects [13,14,15]. In an epidemiological study of adults, hypertension risk was 8% lower in the quintile of the highest anthocyanin intake [16]. Similarly, high anthocyanin intake was associated with a 25% reduced risk of coronary artery disease in two cohort studies of middle-aged men and women, and a 32% reduced risk of myocardial infarction in one cohort study of young and middle-aged women [17]. In a randomized controlled trial, blueberry intake improved insulin sensitivity by approximately 22% in obese individuals with insulin resistance [18]. Similar findings were also reported from our group following strawberry supplementation in adults with features of Metabolic Syndrome (MetS) [19]. Further, a meta-analysis reported significant improvements in high-density lipoprotein cholesterol, low-density lipoprotein cholesterol (LDL-C), and triglyceride concentrations following berry consumption [20]. These data suggest that dietary berries have a protective effect against cardiometabolic risk factors in adults.

Oxidative stress underlies the broad spectrum of cardiometabolic conditions [21,22]. Although evidence has linked dietary berry intake to improved cardiometabolic risk factors, the relationship between berries and antioxidant status in this condition is less clear. To our knowledge, there is no reported systematic review or meta-analysis that investigates the effect of dietary berries on antioxidant biomarkers in adults with cardiometabolic risks. Thus, we have conducted the present systematic review to investigate these effects and assess the magnitude of changes caused by dietary berry consumption on oxidative stress and antioxidant biomarkers in clinical trials.

## 2. Methods

We conducted the present systematic review according to the Preferred Reporting Items for Systematic Reviews and Meta-Analyses (PRISMA) 2020 statement [23]. This review is registered in the International Prospective Register of Systematic Reviews (PROSPERO 2022) as CRD42022374654.

### 2.1. Eligibility, Information Sources, and Search Strategy

Eligible studies met the following inclusion criteria: (1) randomized controlled trial design, (2) human model, (3) adults with cardiometabolic risk factors, (4) supplementation with dietary berries in any form, and (5) antioxidant or oxidative stress outcome measures. Exclusion criteria included the following: (1) animal or in vitro models, (2) adolescents (under 18 years of age), (3) healthy adults without cardiometabolic conditions (e.g., average body mass index in normal range), (4) supplementation with botanical berries not commonly recognized as dietary berries (e.g., blackcurrants, pomegranates, grapes), and (5) non-antioxidant or oxidative stress outcome measures.

Studies included in this review were identified from Medline (accessed by PubMed), Cochrane Library, and Web of Science. The search in Medline used the following filters: “clinical study”; “clinical trial”; “clinical trial phase I, II, III, IV”; “randomized controlled trial”; “humans”; “English”; “adult”. The search in Cochrane Library used the “trials” filter, and filters were not applied in the Web of Science search. The reference lists of each included study were screened manually to identify other studies that met the inclusion criteria. We concluded the search of databases on 25 November 2022 and finished the screening of references on 4 February 2023.

The following search terms were used in all databases: “strawberries”, “cranberries”, “blueberries”, “lingonberries”, “berries”, “raspberries”, “lipid peroxidation”, “catalase”, “glutathione”, “cardiometabolic”, “antioxidant”, “insulin resistance”, and “metabolic syndrome”.

### 2.2. Selection and Data Collection Process

Studies were assessed first by their title and abstract, and then by the participant’s cardiometabolic health status and berry supplementation. The studies that aligned with the predetermined eligibility criteria were fully reviewed. A single reviewer screened each study retrieved in the search (M.M.H.). An additional reviewer independently reviewed all final studies included (A.B.). The reviewers were not blinded to the study authors, institutions, or manuscript journals.

The two same reviewers performed data extraction independently. For each study, the reviewers collected publication data, study design, participant characteristics, intervention, and time of exposure. The reviewers only collected results data that pertained to antioxidant and/or oxidative stress biomarkers.

### 2.3. Assessment of Risk of Bias in Included Studies

We used the Cochrane Methods’ Risk of Bias 2 tool (2019 version) to determine methodological quality of the effect of assignment for each included study [24]. This tool assessed results related to antioxidant and oxidative stress biomarkers at the end of intervention and follow-up timepoints (if included). The 2019 version of the tool was used for parallel trials [24]. For crossover trials, the Risk of Bias 2 for crossover tool (2021 version) was used and an analysis of both periods were selected [25]. All included articles (n = 54) were split in half to be reviewed by two groups of reviewers (T.A., D.K.Z., K.I., and A.B.) independently using the Risk of Bias 2 tool.

The domains assessed included the following: (1) bias arising from the randomization process, (2) bias due to deviations from intended interventions, (3) bias due to missing outcome data, (4) bias in measurement of the outcome, and (5) bias in selection of the reported result [24]. Signaling questions for each domain allowed for a judgement of “low risk of bias”, “high risk of bias”, or “some concerns”. The excel tool algorithm generated an overall risk of bias judgement based on the ranking of each domain. This overall judgement led to the assessment of the certainty of evidence using the Grading of Recommendations, Assessment, Development, and Evaluations approach [26].

### 2.4. Effect Measures and Synthesis Methods

Studies that met the eligibility criteria were eligible for synthesis based on participant characteristics (either having features of MetS or having obesity/overweight). Between group effect sizes for antioxidant and oxidative stress biomarkers were calculated using Cohen’s *d* for studies that included the necessary data (i.e., mean, standard deviation, sample size). Standard error was converted to standard deviation to complete this calculation when necessary. This review omitted effect size of included studies if the study did not provide the necessary data for the calculation.

## 3. Results

### 3.1. Study Selection and Characteristics

**Figure 1:**
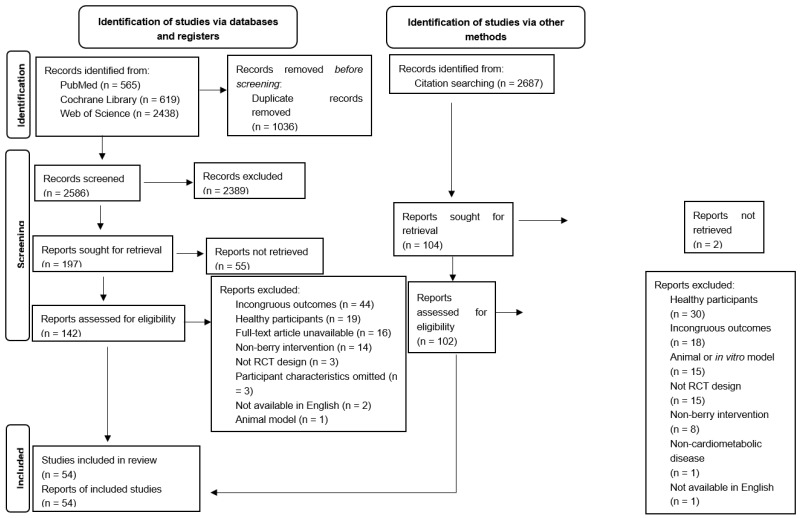
PRISMA flow diagram illustrating the search and selection of studies using the agreed upon inclusion and exclusion criteria.

This search yielded 6299 articles from databases, and after removing duplicates, 2586 articles were screened. An additional 2687 articles were identified through citation searching. In total, 54 studies (~2040 participants) were included in this review (Figure 1). All included studies were randomized controlled trials—30 used a parallel design and 24 used a crossover designed. Overall, 37 studies measured the effects of berries in adults with features of MetS [27,28,29,30,31,32,33,34,35,36,37,38,39,40,41,42,43,44,45,46,47,48,49,50,51,52,53,54,55,56,57,58,59,60,61,62,63] (Table 1) and 17 studies measured these same effects in adults with obesity or overweight [64,65,66,67,68,69,70,71,72,73,74,75,76,77,78,79,80] (Table 2).

### 3.2. Quality Assessment

Using the Risk of Bias 2 tool, we assessed all of the study’s assignment to the intervention. Studies were assessed based on study design as parallel (Table 3) or crossover (Table 4) trials. Both the parallel and crossover studies had some concerns with randomization and the crossover studies had some concern with period and carryover effects. Overall, the majority (61%) of assessed studies had a low risk of bias, 19% had a high risk of bias, and 20% had some concerns.

### 3.3. Adults with Features of MetS

The 37 studies measuring outcomes in adults with features of MetS included 14 different dietary berries. Overall, 21 studies assessed only oxidative stress biomarkers [28,30,31,32,34,36,37,38,40,42,43,45,48,50,51,53,54,56,58,59,60,61], 13 studies assessed both oxidative stress and antioxidant biomarkers [27,29,35,39,41,42,44,46,47,52,55,57,62], and 3 studies assessed only antioxidant biomarkers [33,49,63].

Out of the studies assessing oxidative stress biomarkers, 15 studies reported statistically significant changes in the outcomes [30,31,36,38,43,45,48,50,51,53,54,56,58,59,60,61]. Across the studies, the most common oxidative stress biomarker was oxidized products, in particular oxidized LDL-C. Intake of freeze-dried strawberry powder [59], Korean blackberry [30], freeze-dried blueberry powder [56], cranberry juice [61], and black raspberry [45] reduced levels of oxidized LDL-C, with acute freeze-dried strawberry powder consumption (10 g with a high-fat meal) having the largest effect (*d* = 4.97). These berry interventions reduced plasma-oxidized LDL-C by 9 to 34% from baseline. In addition to oxidized LDL-C, freeze-dried blueberry powder also significantly reduced oxidized purines in blood mononuclear cells with a moderate effect (*d* = 0.60) [53]. Cranberry juice also significantly reduced levels of lipid peroxidation and protein oxidation, however the magnitude of effect cannot be determined due to omitted data from the study [54]. Due to the heterogeneity of the interventions, dose-response relationships between berry intake and oxidized products reductions are challenging to infer.

In addition to oxidized products, biomarkers of oxidative damage were significantly reduced by freeze-dried strawberry powder [50], dried whortleberry [51], and freeze-dried blueberry powder [56]. A low (25 g) and high (50 g) dose of freeze-dried strawberry powder reduced combined malondialdehyde and 4-hydroxy-2-nonenal by approximately 33% from baseline with a very large between group effect (*d* = 2.62 and 7.20, respectively) [50]. A dosage of 500 mg of dried whortleberry reduced malondialdehyde by 12% from baseline with a moderate between group effect (*d* = 0.57) [51]. Because of omitted baseline values, the magnitude of effect of freeze-dried blueberry powder on malondialdehyde and 4-hydroxy-2-nonenal cannot be determined [56]. As a biomarker of lipid peroxidation, concentrations of 8-isoprostanes were statistically significantly reduced from baseline by 31% and 4% by acaí pulp [36] and cranberry powder [31], respectively. Both of these interventions had a modest effect between groups (*d* = 0.33 and 0.42 respectively). Similarly, strawberries [60] and aronia extract [58] reduced thiobarbituric acid reactive substances, a measurement of lipid peroxidation, with a large effect (*d* = 1.36 and 1.25, respectively). With respect to oxidative damage in deoxyribonucleic acid, freeze-dried blueberries reduced hydrogen peroxide damage by 2%, as measured by percent of deoxyribonucleic acid in tail [53]. The blueberry powder had a medium effect on the damage reduction (*d* = 0.85) [53]. These data suggest that strawberry intake has the greatest effect of reducing peroxidation in cells and blueberry intake has protective effects on nucleic acid oxidative damage.

Antioxidant enzymatic activity was also assessed to determine the effect of dietary berries in neutralizing reactive oxygen species. Intake of 100 mg of aronia extract for two months significantly increased serum superoxide dismutase (29% from baseline) and decreased serum catalase (18% from baseline) [58]. The intervention had a very large between group effect on superoxide dismutase (*d* = 2.18) and a large effect on catalase (*d* = 1.33) [58]. Freeze-dried blueberry powder for two months also increased serum superoxide dismutase by 138%, however the study did not provide between group statistical analysis and thus the magnitude of effect cannot be calculated [48]. Interestingly, serum superoxide dismutase also increased by 113% in the group of participants receiving the macronutrient-matched placebo powder [48]. The statistically significant increase within the intervention and comparison groups suggests the increase in enzymatic activity may not be related to the berry intervention. Whole blood and monocyte superoxide dismutase production rates, on the other hand, statistically significantly reduced with consumption of freeze-dried blueberries [38]. Wolfberry extract also reduced erythrocyte superoxide dismutase and increased erythrocyte catalase activities [43]. The magnitude of effect from the freeze-dried blueberry [38] or wolfberry [43] interventions cannot be calculated due to data omitted in each study. Based on these findings, aronia extract appears to have the greatest effect on serum oxidative stress enzymatic activity but quantifying enzymatic activity in blood cells is a challenge.

Similar to the studies that exclusively assessed oxidative stress, the studies that assessed biomarkers in both categories of outcomes predominately measured oxidized products and oxidative damage in addition to antioxidant capacity. Eight weeks of cranberry juice consumption reduced oxidized LDL-C by 33%, combined malondialdehyde and 4-hydroxy-2-nonenal by 50%, and increased plasma antioxidant capacity by 47% from baseline [55]. Compared to the placebo group, the intervention had a moderate to very large effect on each of these outcomes (*d* between 0.67 to 2.06) [55]. Acute consumption of freeze-dried strawberry powder also statistically significantly reduced oxidized LDL-C but did not have an impact on total antioxidant capacity quantified by oxygen radical absorbance capacity [47]. These data suggest that cranberries may have a more comprehensive effect on protecting against oxidative stress by reducing oxidized products and increasing antioxidant capacity.

In addition to antioxidant capacity, three studies also measured enzymatic activity related to oxidative stress [27,39,46]. Low dose (25 g) and high dose (50 g) of freeze-dried strawberry powder consumed for 12 weeks statistically significantly increased plasma antioxidant capacity with an immense effect (*d* = 4.33 and 7.60, respectively) [46]. The doses resulted in an 81% and 72% increase in antioxidant capacity from baseline, respectively [46]. Both doses of the intervention also immensely increased serum whole blood glutathione reductase (*d* = 4.61 and 10.75, respectively), while only the low dose had a significant impact on serum catalase (76% increase) with a large effect (*d* = 2.07) [46]. Freeze-dried strawberry powder at a low dose (13 g) and high dose (32 g) consumed for four weeks also statistically significantly increased serum antioxidant capacity [27]. Interestingly, these doses had lower antioxidant capacity increases from baseline (25% and 40%, respectively) and only had a moderate effect on the outcome (*d* = 0.37 and 0.62, respectively) [27]. These results suggest that longer exposure to the intervention amplifies the improvements in serum antioxidant capacity. These doses of freeze-dried strawberry powder also increased serum superoxide dismutase by 100% and 200%, respectively [27]. The 32 g dose had a larger effect on this outcome (*d* = 1.00 versus 0.51) [27]. Erythrocyte superoxide dismutase, on the other hand, statistically significantly decreased with consumption of goji berries [39]. Consumption of goji berries also reduced erythrocyte catalase and increased serum antioxidant capacity and blood reduced glutathione [39]. The magnitude of effect of the goji berries, however, cannot be calculated based on the data provided in the study [39].

Two studies assessed the effects of different species of blueberries on oxidative stress in nucleic acids in addition to antioxidant biomarkers [35,42]. A 22 g blend of highbush and Rubel blueberries had an immense effect (*d* = 4.54) on reducing 8-hydroxy-2′-deoxyguanosine (13% reduction from baseline), however the reduction was not sustained from week four to week eight [42]. This blend of blueberries did not have any statistically significant impact on serum glutathione reductase [42]. Andean blueberries, on the other hand, only had a moderate effect on reducing 8-hydroxy-2′-deoxyguanosine (*d* = 0.54) but did increase antioxidant capacity as measured using the 2,2-diphenyl-1-picrylhydazyl method [35]. The Andean blueberry effect on antioxidant capacity was small (*d* = 0.31). The findings from these two studies suggest that consuming these species of blueberries for increasing antioxidant capacity may have limited practical meaningfulness.

Finally, neither chokeberry [62] nor bilberry [57] juice improved biomarkers of lipid peroxidation, but both did increase plasma availability of antioxidants. Chokeberry juice increased concentration of vitamin A with a large effect (*d* = 0.94) [62] and bilberry juice increased plasma quercetin and *p*-coumaric acid [57]. The magnitude of the bilberry juice’s effect cannot be calculated due to omitted data in the study. Of the studies that only assessed antioxidant outcomes, none reported statistically significant findings.

### 3.4. Adults with Obesity or Overweight

The 18 studies measuring outcomes in adults with obesity or overweight included 6 different dietary berries. Five studies assessed both oxidative stress and antioxidant biomarkers [64,65,73,76,78], six studies assessed only antioxidant outcomes [67,70,74,77,79,80], and six studies assessed only oxidative stress biomarkers [66,68,69,71,72,75].

Among the studies assessing both categories of biomarkers, three reported significant changes in the outcomes [64,73,78]. A 250 mg and 500 mg dosage of Indian gooseberry increased serum glutathione from baseline by 24% and 53%, respectively, after 12 weeks of supplementation [64]. Between groups, the 250 mg intervention had a large effect on plasma glutathione (*d* = 0.99) and the 500 mg intervention had a very large effect (*d* = 2.79). Similarly, 500 mg of raspberry ketone supplementation for 12 weeks yielded a 26% increase in serum glutathione from baseline [73]. The raspberry ketones had a large effect this increase (*d* = 1.37) in between group analysis [73]. Both Indian gooseberries and raspberry ketones also yielded a statistically significant decrease in serum malondialdehyde. The 250 mg dosage of Indian gooseberries decreased serum malondialdehyde by 21% with a large effect (*d* = 1.69) and the 500 mg dosage decreased the biomarker by 31% with a very large effect (*d* = 2.90) [64]. The 500 mg of raspberry ketones decreased the biomarker by 43%; however, the intervention only had a medium effect (*d* = 0.42) [73]. These results suggest similar effectiveness between Indian gooseberry and raspberry ketones on serum glutathione and malondialdehyde after 12 weeks in adults with overweight [64] and obesity [73]. The third study measured plasma F_2_-isoprostanes, lipid hydroperoxides, and total plasma antioxidant potential after three weeks of consuming 250 mg of blueberries [78]. This dosage of blueberries decreased plasma concentration of lipid hydroperoxides by 50% from baseline [78]. The article omitted necessary data to determine between group change or effect size.

Of the studies assessing only antioxidant biomarkers, five reported statistically significant changes in the outcomes [70,74,77,79,80]. Indeed, 10, 20, and 40 g of freeze-dried strawberry powder yielded statistically significant increases in peak plasma anthocyanin concentration [74]. Pelargonidin-*O-*glucuronide increased by 93, 167, and 226 nmol/L, respectively, with exceptionally large effect sizes (*d* = 6.74, *d* = 4.66 *d* = 12.02, respectively). The reported 11, 13, and 16 nmol/L increase in pelargonidin-3-*O*-glucoside did not statistically differ between 10, 20, and 40 g of supplementation. Similarly, the reported 4 and 5 nmol/L increase in cyanidin-3-*O*-glucoside from 10 and 20 g supplementation, respectively, did not statistically differ from one another, but the 40 g supplementation yielded a statistically significant increase of 7 nmol/L with a large effect (*d* = 3.87). A second study reported similar results with 34 g of freeze-dried strawberry powder statistically significantly increasing plasma pelargonidin sulfate and pelagonidin-3-*O*-glucoside by approximately 15% and 86% compared to the comparison group [77]. The study omitted data necessary to calculate effect size of the intervention. A third study also reported statistically significant increases in plasma phenolic compounds after fresh strawberry consumption [79]. The magnitude of change and effect of the intervention compared to the comparison group cannot be calculated due to omitted data in the article. That said, these three studies suggest a high bioavailability of fresh and freeze-dried strawberries that result in increased plasma antioxidant biomarkers in individuals with obesity and overweight.

The other two studies that assessed only antioxidant biomarkers reported statistically significant changes in antioxidant capacity using multiple methods (2,2-diphenyl-1-picryl-hydrazyl scavenging capacity [74], ferric reducing ability of plasma [74,80], and oxygen radical absorbance capacity [80]). A beverage with 240 g of fresh strawberries increased oxygen radical absorbance capacity and ferric reducing ability of plasma by 12% and 10%, respectively, compared to a control beverage four hours after consumption [80]. The intervention had a moderate effect on the increase in oxygen radical absorbance capacity and ferric reducing ability of plasma (*d* = 0.84 and *d* = 0.61, respectively). Ferric reducing ability of plasma and non-urate ferric reducing ability of plasma also statistically significant increased after seven-day consumption of 500 g of fresh strawberries [80]. Compared to fasting values, non-urate ferric reducing ability of plasma increased by 26% after strawberry consumption [80]. The strawberry consumption also yielded a statistically significant decrease in 2,2-diphenyl-1-picryl-hydrazyl scavenging capacity on the eleventh and fourteenth day of consumption compared to baseline (44% and 40%, respectively) [80]. Data to determine effect size differences between groups was not provided. Although effect of the intervention could not be calculated for both studies, these results suggest increased antioxidant capacity from the consumption of fresh strawberries in overweight adults.

An additional study that assessed plasma antioxidant outcomes did not provide statistical analysis of results but reported concentration of anthocyanins and phenolic acids across 24 h after consumption of 25 g freeze-dried strawberries [67]. Zhong et al. reported peonidin glycosides having the highest fractional bioavailability, vanillic acid glucuronide having the highest concentration, and a biphasic response of anthocyanin glucuronide metabolites [67].

In the studies assessing only oxidative stress outcomes, two reported statistically significant decreases in the related biomarkers [66,72]. Consumption of 50 g of freeze-dried strawberry powder reduced serum 4-hydroxynonenal-2-nonenal-modified proteins, however data are not available to calculate the magnitude of effect [66]. The second study reported reductions in oxidized LDL-C and urinary F_2_-isoprostanes compared to baseline after consumption of 150 mg maqui berry extract [72]. Similar to the previously mentioned study, data are not available to calculate the magnitude of effect. Both of these studies suggest protective implications from berry consumption on oxidative stress, but the strength of the relationship cannot be determined based on the available data.

### 3.5. Secondary Outcomes

In addition to biomarkers of antioxidant status and oxidative stress, some studies assessed features of the MetS (i.e., triglycerides, blood pressure, blood glucose, waist circumference, and high-density lipoprotein cholesterol). One study reported the prevalence of MetS after the intervention, and daily consumption of agraz nectar resulted in 22.5% fewer women having at least three MetS criteria after four weeks; thus, they no longer had MetS [35]. This study, however, did not report specificity on the metabolic features that were reduced in the women who no longer had MetS after the intervention [35].

Whortleberry [51], chokeberry [62], aronia extract [58], and gooseberry [64] reduced triglyceride concentrations by 18.5 mg/dL [64] to 49.6 mg/dL [51]. Gooseberry yielded a dose-dependent reduction with the larger dosage (500 mg versus 250 mg) resulting in a 76% greater reduction [64]. Similarly, chokeberry [62] and aronia extract [58] reduced diastolic and systolic blood pressure by 5–7 mmHg and 11.5–13.5 mmHg, respectively. Freeze-dried blueberry powder yielded a similar reduction in diastolic blood pressure [48], but a lesser reduction in systolic blood pressure (7 mmHg) [48,56]. Chokeberry consumption also reduced serum glucose by 7.7 mg/dL [62]. Of the final two criteria for MetS, waist circumference was reduced by goji berry [39], raspberry ketones [73], and cranberry juice [63] by 6 cm, 4 cm, and 2 cm, respectively. Cranberry juice [63] and aronia extract [58] both increased high-density lipoprotein cholesterol by approximately 1.5 mg/dL. The increase from the aronia extract, however, was not great enough to surpass 40 mg/dL which is the minimum concentration of high-density lipoprotein cholesterol to not meet MetS features. Even though not all the studies assessed each criterion of MetS, these results suggest that chokeberry may be the most effective in modulating features of MetS.

## 4. Discussion

The results of our systematic review documented a range of effectiveness of berry interventions in addressing oxidative stress and antioxidant biomarkers in adults. In individuals with features of MetS and in individuals with overweight or obesity, the percentage of reported significant improvements were similar (62% and 58% of studies). Consumption of 50 g of freeze-dried strawberries for 12 weeks had the largest effect on these outcomes, specifically increasing antioxidant capacity (*d* = 7.60) and whole blood glutathione reductase (*d* = 10.75), in adults with obesity and elevated serum lipids [46]. This dosage of freeze-dried strawberries also had large effects on decreasing combined levels of malondialdehyde and 4-hydroxynonenal (*d* = 7.20) in hyperlipidemic adults [50]. Multiple interventions also significantly reduced oxidized LDL in adults with features of MetS (within and between group analysis); however, due to omitted data the magnitude of effect could not be calculated. In overweight adults, 12-week supplementation of 1000 mg of Indian gooseberry largely increased levels of glutathione (*d* = 2.79) and decreased levels of malondialdehyde (*d* = 2.90) [64]. Various doses of freeze-dried strawberry powder also substantially increased antioxidant activity as measured by phenolic compounds in adults with obesity (*d* between 3.06 and 12.02) [74]. Collectively, the berry interventions in adults with overweight or obesity had less significant effects on oxidative stress biomarkers.

Each of the features of MetS—hypertension, hyperglycemia, dyslipidemia, and abdominal obesity [81]—have some relationship with oxidative stress. Animal models have demonstrated a causal relationship between nitrogen oxides and hypertension induced by angiotensin II [82]. In mice deficient of nicotinamide adenine dinucleotide oxidase 1, vascular superoxide production and gradual blood pressure increase were stunted in the aorta after infusion of angiotensin II [83]. In wild type mice, however, this infusion increased blood pressure and vascular superoxide production [83]. A similarly designed study also produced these results in addition to reduced media hypertrophy [84], supporting the relationship between nicotinamide adenine dinucleotide oxidase-generating reactive oxygen species and induced hypertension. Increased and dysregulated blood glucose levels increase biomarkers of oxidative stress through various pathways such as mitochondrial mechanisms, cellular antioxidant systems, and lipid peroxidation [85]. Hyperglycemia glycates metabolic end products in the extracellular matrix which bind with certain receptors to increase the production of reactive oxygen species [86]. In addition, glycation effects enzymatic activity which is demonstrated in reduced activity of catalase and superoxide dismutase in diabetic rats compared to control rats [87]. Finally, visceral adiposity is positively associated with dyslipidemia due to increased plasma free fatty acids [88,89]. Both of these features increase oxidative stress by activating reduced nicotinamide adenine dinucleotide phosphate, and animal models have indicated induction of this pathway by white adipose tissue [90].

In addition to the relationship between MetS and oxidative stress, MetS was also reported to decrease antioxidant status [91]. Thus, the findings of the present review may play an influential role in improving health outcomes in individuals with MetS. Glutathione reductase modulates reactive oxygen species by increasing antioxidant activity [92], malondialdehyde and 4-hydroxynonenal are both byproducts of lipid peroxidation [93], and oxidized LDL-C is atherogenic [94] and strongly associated with MetS [95,96,97]. Serum levels of glutathione are 30 to 60% less in individuals with MetS [91,95]. Thus, increasing glutathione reductase may modulate the decreased antioxidant status by increasing the supply of reduced glutathione. Improvements in antioxidant activity and reductions in lipid peroxidation byproducts may improve an individual’s blood pressure, blood glucose levels, and lipid metabolism. In a longitudinal study, dietary antioxidant capacity was negatively correlated to hypertension and increases in capacity reduced risk of abdominal obesity by 38% [98]. Thus, the findings from this review provide meaningful mechanisms to address the decreased antioxidant status and increased oxidative stress found in individuals with features of MetS.

The reported results in individuals with overweight or obesity similarly reflect the relationship between adiposity and oxidative stress. Adiposity diminishes plasma redox status by nearly 50%, reduces serum levels of glutathione by 26%, and increases advanced glycated end products by 23% compared to healthy controls [91]. Animal models also confirm reduced antioxidant capacity in obesogenic conditions with 30% reduced total antioxidant status and reduced erythrocyte catalase and superoxide dismutase activity compared to control rats [99]. Similarly, plasma hydroperoxide levels are 48% higher in obese rats than in control rats. The findings from this review suggest promising interventions such as freeze-dried and fresh strawberries increasing plasma antioxidant compounds [74,79], raspberry ketones increasing glutathione levels [73], and modest increases in antioxidant capacity from fresh strawberries [80].

The certainty of evidence from this review is dependent on the study design. The parallel studies were more robust with limited missing outcome data and low concerns of measurement of outcomes and reported results. The findings from these studies represent the true treatment effects since the majority of studies had a low overall risk of bias. That said, the crossover studies had a higher risk of bias with concerns related to carryover effects, deviation from the intervention, and selection of reported results. The effects of the crossover studies should be interpreted with caution due to these concerns. For both study designs, more information from the authors about random sequence allocation and baseline participant data would have improved randomization-related bias. For the crossover trials, study designs could have been improved with justification of the time of the washout period, the inclusion of period effects in the analysis and reporting of all eligible results.

Although most studies had a low risk of bias and the parallel trials had a robust study design allowing for strong certainty of evidence, there are methodological limitations to the studies included in this review. The included studies do not address sustained improvements of oxidative stress and antioxidant biomarkers. The results may not be maintained after the exposure time or plateau. In addition, while all the participants had at least one cardiometabolic risk factor, the heterogeneity of characteristics limits the generalizability of the findings broadly to any cardiometabolic risk factor. The favorable effects reported in this review may be specifically linked to each group of participants’ risk factors (e.g., dyslipidemia compared to obesity). The studies also had heterogeneity in berries used, dosage, and time of exposure. The difference in dosage and time of exposure challenges the ability to make meaningful dietary recommendations based on these findings. Despite these limitations, the present review followed the structured and focused search and selection processes as defined by the PRISMA 2020 statement, which strengthens the evaluation of the findings [23].

## 5. Conclusions

**Figure 2:**
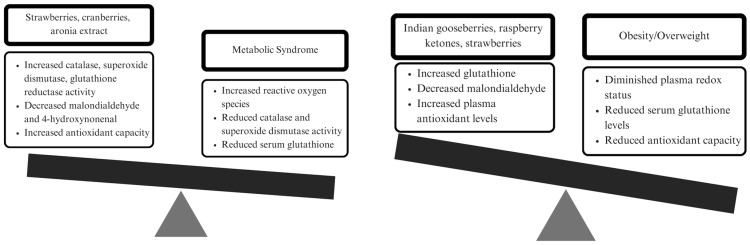
Graphic depiction of the effects of certain dietary berries on oxidative stress and antioxidant status biomarkers in adults with Metabolic Syndrome versus obesity/overweight.

In conclusion, the effect of berry intake on oxidative stress and antioxidant status in individuals with cardiometabolic risk factors is promising, but inconsistent across berry type and exposure time. Based on the included studies, berries yield greater effects on oxidative stress biomarkers in individuals with features of MetS compared to those with only overweight or obesity (Figure 2). Berries did positively affect antioxidant capacity in both sets of participants. Due to the quality of study design, the evidence from the parallel trials is stronger than that of crossover trials, thus some results should be analyzed with caution. That said, clinical practice and public health nutrition approaches can still incorporate the findings of this review as the promotion of berry intake aligns with standard nutrition recommendations (Table 5). Future investigations should address the concerns mentioned related to quality of study design and incorporate long-term follow-up to assess sustained effects of berry intake.

## Figures and Tables

**Table 1:** Effects of berries on antioxidant and oxidative stress biomarkers in adults with features of Metabolic Syndrome.

Author, Year (Country)	RCT Design	Participant Age (±SD) and Sex	Participant Cardiometabolic Risk Factor	Intervention	Time of Exposure	Plasma/Serum *(unless otherwise stated)* Antioxidant and/or Oxidative Stress Outcomes	Between Group Significant Outcomes *(unless otherwise stated)* (Cohen’s *d, 95% CI*)
Basu et al., 2021 (USA) [27]	Double-blinded, crossover (*n* = 33)	53 ± 13 years 2M/31F	Obesity; elevated LDL-C	Intervention 1: 13 g FDS powder Intervention 2: 32 g FDS powder	Powder divided into 2 servings per day for 4 weeks	Catalase; GSH; GSH reductase; reduced GSH; SOD; peroxidase; TAC; MDA	13 g: ↑ SOD (0.51) ↓ MDA (0.51) ↑ TAC (0.37) 32 g: ↑ SOD (1.00) ↓ MDA (0.73) ↑ TAC (0.62)
Richter et al., 2021 (USA) [28]	Double-blinded, crossover (*n* = 40)	47 ± 12 years 25M/15F	Overweight; SBP ≥ 120 mmHg and/or DBP ≥ 80 mmHg	500 mL cranberry juice	250 mL of juice twice per day for 4 weeks	Isoprostanes	∅
Marin-Echeverri et al., 2021 (Colombia) [29]	Double-blinded, crossover (*n* = 40)	47 ± 9 years 40F	Classified with MetS	7.38 g lyophilized agraz per 200 mL water (~200 g fresh agraz)	200 mL of beverage per day for 4 weeks	SOD; catalase; GPx; ABTS; FRAP; ORAC; 8-OHdG; F2-isoprostane	∅
Cho et al., 2020 (South Korea) [30]	Double-blinded, parallel (*n* = 77)	Control: 48 ± 12 years 6M/32F Intervention: 47 ± 12 years 13M/26F	Total blood cholesterol between 200–239 mg/dL	600 mg freeze-dried Korean blackberry	Consumed daily for 12 weeks	Ox-LDL	↓ ox-LDL (0.69)
Hsia et al., 2020 (USA) [31]	Double-blinded, parallel (*n* = 37)	Control: 48 ± 12 years 4M/13F Intervention: 47 ± 16 years 8M/8F	Obesity; elevated FPG or impaired glucose tolerance; insulin concentration ≥ 5 μIU/mL	0.062 g cranberry powder per 100 mL beverage	450 mL of beverage per day for 8 weeks	Ox-LDL; lectin-like ox-LDL receptor 1; 8-isoprostane; MDA; advanced glycated end products; paraoxonase-1	↓ 8-isoprostane (0.42)
Xiao et al., 2019 (USA) [32]	Single-blinded, 3-arm, crossover (*n* = 32)	34 ± 12 years 17M/15F	Elevated FPG and fasting insulin; HOMA-IR ≥ 2.5	Intervention 1: 125 g frozen red raspberries Intervention 2: 250 g frozen red raspberries	Consumed with 900 kcal challenge meal typical of Western eating patterns	Ox-LDL	∅
Quintero-Quiroz et al., 2019 (Colombia) [33]	Double-blinded, crossover (n = 66)	47 ± 10 years 26M/40F	Classified with MetS	7.38 g lyophilized agraz per 200 mL water (~200 g fresh agraz)	200 mL of beverage per day for 4 weeks	FRAP; DPPH scavenging activity	∅
Marin-Echeverri et al., 2018 (Colombia) [34]	Double-blinded, crossover (*n* = 40)	47 ± 9 years 40F	Classified with MetS	Freeze-dried agraz reconstituted in 200 mL water (~200 g fresh agraz)	200 mL of beverage per day for 4 weeks	PON1 arylesterase and lactonase activity; MPO; AOPP	∅
Espinosa-Moncada et al., 2018 (Colombia) [35]	Double-blinded, crossover (*n* = 40)	47 ± 9 years 40F	Classified with MetS	200 mL agraz nectar	200 mL of beverage per day for 4 weeks	Antioxidant capacity; TBARS; F_2_-isoprostanes; urinary 8-OHdG	↑ antioxidant capacity (0.31) ↓ urinary 8-OHdG (0.54)
Kim et al., 2018 (USA) [36]	Double-blinded, parallel (*n* = 37)	Control: 42 ± 14 years Intervention: 47 ± 12 years 11M/26F	Classified with MetS	12% solid açaí pulp (~81 g açaí pulp)	325 mL beverage twice per day for 12 weeks	8-isoprostane	↓ 8-isoprostane (0.33)
Feresin et al., 2017 (USA) [37]	Double-blinded, parallel (*n* = 60)	45–65 years 60F	Pre- or stage-1 hypertension	Intervention 1: 50 g FDS powder Intervention 2: 25 g FDS powder	Powder consumed once per day for 8 weeks	SOD	∅
Nair et al., 2017 (USA) [38]	Double-blinded, parallel (*n* = 27)	Control: 59 ± 3 years 2M/10F Intervention: 55 ± 2 years 7M/8F	Classified with MetS	22.5 g freeze-dried blueberry powder	12oz yogurt and skim-milk based smoothie twice per day for 6 weeks	Whole blood and monocyte total ROS; whole blood and monocyte superoxide production rates	↓ whole blood and monocyte ROS and superoxide production (NP)
Zanchet et al., 2017 (Brazil) [39]	Parallel (*n* = 50)	Control: 49 ± 13 years 7M/18F Intervention: 53 ± 11 years 8M/17F	Classified with MetS	14 g goji berry	Natural form consumed daily for 45 days	FRAP; reduced GSH; MDA; erythrocyte catalase; erythrocyte SOD; TBARS	All within group: ↑ FRAP (NP) ↑ TBARS (NP) ↑ GSH (NP) ↑ catalase (NP) ↓ SOD (NP)
Stote et al., 2017 (USA) [40]	Single-blinded, crossover (*n* = 19)	53 ± 6 years 20F	Two risk factors for T2DM	240 mL wild blueberry juice	Half of dosage consumed twice per day for 1 week	Ox-LDL; 8-isoprostane	∅
Paquette et al., 2017 (Canada) [41]	Double-blinded, parallel (*n* = 41)	Control: 60 ± 5 years 9M/12F Intervention: 57 ± 4 years 9M/11F	Overweight or obesity; fasting plasma insulin > 60 pmol/L	1.84 g mix of dry strawberry and cranberry polyphenol extracts	Beverage consumed daily for 6 weeks	Ox-LDL; FRAP	∅
Johnson et al., 2017 (USA) [42]	Double-blinded, parallel (*n* = 40)	Control: 57 ± 5 years Intervention: 60 ± 5 years 40F	Obesity; pre- or stage-1 hypertension	22 g freeze-dried blueberry powder (~1 cup fresh blueberries)	11 g with 240 mL of water twice per day for 8 weeks	SOD; 8-isoprostane; GPx; GSH reductase; ox-LDL; DNA 8-OHdG	4 weeks: ↓ 8-OHdG (4.54) 8 weeks: ∅
Lee et al., 2017 (Korea) [43]	Double-blinded, parallel (*n* = 53)	Control: 52 ± 8 years 3M/24F Intervention: 50 ± 7 years 9M/17F	Overweight; LDL-C between 130–165 mg/dL	13.5 g wolfberry extract	Beverage consumed daily for 8 weeks	Erythrocyte SOD, catalase, GPx; ox-LDL; MDA	↓ SOD (NP) ↑ catalase (NP)
Xie et al., 2017 (USA) [44]	Double-blinded, parallel (*n* = 49)	Control: 37 ± 15 years 11M/13F Intervention: 33 ± 13 years 13M/12F	Overweight; total serum cholesterol > 200 mg/dL; LDL-C > 100 mg/dL	500 mg aronia berry extract	500 mg daily for 12 weeks	Catalase; GPx; SOD; TAC; 8-isoprostanes	∅
An et al., 2016 (Korea) [45]	Double-blinded, parallel (*n* = 44)	Control: 58 ± 8 years 4M/9F Intervention 1: 60 ± 9 years 4M/10F Intervention 2: 58 ± 7 years 5M/12F	Impaired fasting glucose between 100–125 mg/dL or OGTT between 140–200 mg/dL	Intervention 1: 900 mg black raspberry extract Intervention 2: 1800 mg black raspberry extract	Half of dosage consumed twice per day for 12 weeks	Ox-LDL	↓ ox-LDL (within group, NP)
Basu et al., 2016 (USA) [46]	Parallel (*n* = 60)	49 ± 10 years 5M/55F	Obesity; elevated serum lipids	Intervention 1: 25 g FDS powder Intervention 2: 50 g FDS powder	Half of dosage consumed as beverage twice per day for 12 weeks	Antioxidant capacity; whole blood GSH reductase; catalase; GPx; GSH reductase	Intervention 1: ↑ antioxidant capacity (4.33) ↑ catalase (2.07) ↑ whole blood GSH reductase (4.61) Intervention 2: ↑ antioxidant capacity (7.60) ↑ whole blood GSH reductase (10.75)
Park et al., 2016 (USA) [47]	Single-blinded, 4-arm, crossover (*n* = 21)	40 ± 14 years 5M/16F	Waist circumference > 110 cm; FPG between 5.5–6.9 mmol/L or fasting insulin > 75th percentile cutoff or HOMA-IR ≥ 1.0	Intervention 1: 10 g FDS powder Intervention 2: 20 g FDS powder Intervention 3: 40 g FDS powder	Consumed within 20 min after breakfast meal typical of Western eating patterns	Ox-LDL; ORAC	Intervention 2: ↓ ox-LDL (NP) Intervention 3: ↓ ox-LDL (NP)
Johnson et al., 2015 (USA) [48]	Double-blinded, parallel (*n* = 40)	Control: 57 ± 5 years Intervention: 60 ± 5 years 40F	Blood pressure between 125/85 and 160/90 mmHg	22 g freeze-dried blueberry powder	Consumed daily for 8 weeks	SOD	↑ SOD (within group, NP)
McAnulty et al., 2014 (USA) [49]	Parallel (*n* = 25)	Control: 40 ± 13 years Intervention: 46 ± 12 years NP	Blood pressure ≥ 120/80 mmHg	19 g blueberry powder	Consumed twice per day for 6 weeks	ORAC; FRAP	∅
Basu et al., 2014 (USA) [50]	Parallel (*n* = 60)	49 ± 10 years 5M/55F	Obesity; elevated serum lipids	Intervention 1: 25 g FDS powder Intervention 2: 50 g FDS powder	Half of dosage consumed as beverage twice per day for 12 weeks	Combined MDA and HNE	Intervention 1: ↓ MDA and HNE (2.62) Intervention 2: ↓ MDA and HNE (7.20)
Soltani et al., 2014 (Iran) [51]	Double-blinded, parallel (*n* =50)	Control: 46 ± 17 years 10M/15F Intervention: 48 ± 16 years 10M/15F	Hyperlipidemic	500 mg dried whortleberry	Consumed twice per day for 4 weeks	MDA	↓ MDA (0.57)
Puupponen-Pimiä et al., 2013 (Finland) [52]	Parallel (*n* = 32)	Control: 50 ± 7 years 3M/9F Intervention: 53 ± 7 years 10M/10F	Classified with MetS	100 g strawberry purée 100 g frozen raspberries 100 g frozen cloudberries	Consumed daily for 8 weeks	8-isoprostanes; TRAP	∅
Riso et al., 2013 (Italy) [53]	Repeated-measures, crossover (*n* = 18)	48 ± 10 years 18M	Overweight; one CVD risk factor	25 g freeze dried wild blueberry powder	Beverage consumed daily for 6 weeks	Reduction in oxidized purines; H_2_O_2_-induced DNA damage; reduced GSH; oxidized GSSG; GST; SOD; GPx	↓ oxidized purines (0.60) ↓ H_2_O_2_ damage (0.85)
Simão et al., 2013 (Brazil) [54]	Parallel (*n* = 56)	Control: 49 years 8M/28F Intervention: 51 years 6M/14F	Classified with MetS	0.7 L reduced-calorie cranberry juice	Consumed twice per day for 60 days	Lipo-peroxidation; protein oxidation	↓ lipo-peroxidation (NP) ↓ protein oxidation (NP)
Basu et al., 2011 (USA) [55]	Double-blinded, parallel (*n* = 36)	52 ± 8 years 36F	Classified with MetS	240 mL reduced-calorie cranberry juice	Consumed daily for 8 weeks	Ox-LDL; MDA and HNE; antioxidant capacity	↓ ox-LDL (0.67) ↓ MDA and HNE (2.06) ↑ antioxidant capacity (1.59)
Basu et al., 2010 (USA) [56]	Single-blinded, parallel (*n* = 48)	Control: 48 ± 3 years 2M/21F Intervention: 52 ± 3 years 2M/23F	Obesity; classified with MetS	50 g freeze-dried blueberry powder	Half of dosage consumed as beverage twice per day for 8 weeks	Ox-LDL; MDA and HNE; MPO	↓ ox-LDL (NP) ↓ MDA and HNE (NP)
Karlsen et al., 2010 (Norway) [57]	Parallel (*n* = 62)	Control *: 53 years 25M/7F Intervention: 53 years 21M/10F	Overweight; one CVD risk factor	330 mL bilberry juice	Consumed daily	FRAP; TRAP; ORAC; vitamin C; DHAA; TAA; oxidized GSH; tocopherols; carotenoids; quercetin; lipid peroxidation; oxidized vitamin C	↑ Quercetin (NP) ↑ *p*-coumaric acid (NP)
Broncel et al., 2010 (Poland) [58]	Parallel (*n* = 47)	42–65 years 15M/32F	Classified with MetS	100 mg aronia extract	Consumed three times per day for 2 months	Erythrocyte SOD, catalase, GPx, TBARS	↑ SOD (2.18) ↓ catalase (1.33) ↓ TBARS (1.25)
Burton-Freeman et al., 2010 (USA) [59]	Single-blind, crossover (*n* = 24)	51 ± 15 years 10M/14F	Overweight; hyperlipidemic	10 g FDS powder	Acute: Beverage consumed with high-fat challenge meal Chronic: Beverage consumed daily for 6 weeks	Ox-LDL	Acute: ↓ ox-LDL (4.97) Chronic: ∅
Jenkins et al., 2008 (Canada) [60]	Crossover (*n* = 28)	62 ± 1 years NP	Hyperlipidemic	454 g strawberries	Consumed daily for 1 month after 2.5 years of cholesterol-lowering diet	Protein oxidation; TBARS	↓ TBARS (1.36) ↓ TBARS molar ratio of LDL-C (1.02)
Ruel et al., 2008 (Canada) [61]	Single-blinded, crossover (*n* = 30)	51 ± 10 years 30M	Waist circumference ≥ 90 cm; LDL-C between 3.0–5.0 mmol/L	125 mL, 250 mL, 500 mL cranberry juice	Progressive consumption daily for 12 weeks	Ox-LDL	250 mL: ↓ ox-LDL (within group, NP) 500 mL: ↓ ox-LDL (within group, NP)
Skoczyñska et al., 2007 (Poland) [62]	Crossover (*n* = 58)	54 ± 6 years 58M	Total serum cholesterol ≥ 200 mg/dL	250 mL chokeberry juice	Consumed daily for 6 weeks	Lipid peroxides; vitamin A; vitamin E	↑ vitamin A (0.94)
Ruel et al., 2006 (Canada) [63]	Single-blinded, parallel (*n* = 30)	51 ± 10 years 30M	Waist circumference ≥ 90 cm; LDL-C between 3.0–5.0 mmol/L	125 mL, 250 mL, 500 mL cranberry juice	Progressive consumption daily for 12 weeks	TAC	∅

*All biomarkers are plasma or serum measurements unless otherwise stated in the table. All outcomes are reported as between group unless stated in the table as within group. The Cohen’s d is calculated at a 95% confidence interval.* * One participant dropped out of the study, but the sex of the participant was not provided. ↑: increase; ↓: decrease; ∅: no change; μIU: micro-international units; 8-OHdG: oxo-2′-deoxyguanosine; ABTS: 2,2′-azino-bis(3-ethylbenzothiazoline-6-sulfonic acid); AOPP: advanced oxidation protein products; DHAA: dehydroascorbic acid; dL: deciliter; DNA: deoxyribonucleic acid; DPPH: 2,2-diphenyl-1-picryl-hydrazyl; F: female; FDS: freeze-dried strawberry; FPG: fasting plasma glucose; FRAP: ferric reducing ability of plasma; g: gram; GPx: glutathione peroxidase; GSH: glutathione; H_2_O_2_: hydrogen peroxide; HNE: 4-hydroxynonenal; HOMA-IR: homeostatic model assessment of insulin resistance; kcal: kilocalorie; LDL-C: low density lipoprotein cholesterol; M: male; MDA: malondialdehyde; MetS: Metabolic Syndrome; mg: milligram; mL: milliliter; MMP-9: matrix metalloproteinase-9; MPO: myeloperoxidase; NP: not provided due to omitted data; ORAC: oxygen radical absorbance capacity; ox-LDL: oxidized low density lipoprotein cholesterol; oz: ounce; PON1: paraoxonase 1; ROS: reactive oxygen species; SOD: superoxide dismutase; TAA: total ascorbic acid; TAC: total antioxidant capacity; TBARS: thiobarbituric acid reactive substances; TRAP: total radical-trapping antioxidant parameter; years: years.

**Table 2:** Effects of berries on antioxidant and oxidative stress biomarkers in adults with obesity or overweight.

Author, Year (Country)	RCT Design	Participant Age (±SD) and Sex	Participant Cardiometabolic Risk Factor	Intervention	Time of Exposure	Plasma/Serum *(unless otherwise stated)* Antioxidant and/or Oxidative Stress Outcomes	Between Group Significant Outcomes *(unless otherwise stated)* (Cohen’s *d, 95% CI*)
Usharani et al., 2019 (India) [64]	Double-blinded, parallel (*n* = 59)	Control: 57 ± 7 years 14M/4F Intervention 1: 57 ± 9 years 15M/6F Intervention 2: 57 ± 7 years 14M/6F	Overweight; endothelial dysfunction	Intervention 1: 500 mg Indian gooseberry Intervention 2: 1000 mg Indian gooseberry	Half of dosage. Consumed twice per day for 12 weeks	GSH; MDA	250 mg: ↑GSH (0.99) ↓ MDA (1.69) 500 mg: ↑GSH (2.79) ↓ MDA (2.90)
Chew et al., 2019 (USA) [65]	Double-blinded, parallel (*n* = 78)	43 ± 1 years 33M/45F	Obesity	450 mL cranberry extract beverage	450 mL of beverage per day for 8 weeks	Reduced GSH; oxidized GSSG; GPx; SOD; ox-LDL; F_2α_-isoprostanes; urinary 8-OHdG	∅
Basu et al., 2018 (USA) [66]	Crossover (*n* = 17)	57 ± 7 years 4M/13F	Obesity	50 g FDS powder	50 g twice per day for 12 weeks	4-hydroxy-2-nonenal-HNE modified proteins	↓ HNE-modified proteins (NP)
Zhong et al., 2017 (USA) [67]	Single-blinded, crossover (*n* = 12)	29 ± 5 years 6M/6F	Overweight	25 g freeze-dried wild blueberry powder	Consumed with challenge meal	Anthocyanins; phenolic acids	NP
Richter et al., 2017 (USA) [68]	Crossover (*n* = 30)	28 ± 11 years 17M/13F	Obesity	40 g FDS powder	Consumed with 1000 kcal high-fat challenge meal	MDA: ox-LDL	∅
Huang et al., 2016 (USA) [69]	Single-blinded, 3-arm, crossover (*n* = 14)	Control: 27 ± 4 years 7M/3F Intervention: 25 ± 4 years 2M/2F	Overweight	12 g FDS powder	Consumed with high-fat, high-kcal challenge meal typical of Western eating patterns	Ox-LDL	∅
Prymont-Przyminska et al., 2016 (Poland) [70]	Open label, parallel (*n* = 18)	Control: 47 ± 8 years 5M/2F Intervention: 41 ± 11 years 10M/1F	Overweight	500 g strawberries	Consumed daily for 9 days	DPPH scavenging activity; FRAP; total polyphenols	All within group: ↑ non-urate FRAP (NP) ↑ FRAP (NP) ↓ DPPH activity (NP)
Eftekhari et al., 2016 (Iran) [71]	Double-blinded, parallel (*n* = 40)	Control: 42 ± 7 years Intervention: 42 ± 5 years 40F	Overweight	400 mg cranberry	Consumed daily for 8 weeks	MDA	∅
Davinelli et al., 2015 (Italy) [72]	Double-blinded, parallel (*n* = 42)	45–65 years 29M/13F	Overweight	150 mg maqui berry extract	Consumed three times per day for 4 weeks	Ox-LDL; urinary F_2_-isoprostanes	↓ ox-LDL (NP) ↓ urinary F_2_-isoprostanes (NP)
Khazaal et al., 2015 (Iraq) [73]	Single-blinded, parallel (*n* = 60) *	Control: 33 ± 7 years 18F Intervention: 32 ± 6 years 20F	Obesity	500 mg raspberry ketones	Consumed daily for 12 weeks	GSH; MDA; 8-isoprostanes	↑ GSH (1.37) ↓ MDA (0.42)
Banaszewski et al., 2013 (USA) [74]	Single-blinded, crossover (*n* = 5)	1M/4F	Obesity	Intervention 1: 10 g FDS powder Intervention 2: 20 g FDS Intervention 3: 40 g FDS powder	Beverage consumed with challenge meal	Anthocyanins	10 g FDS: ↑ pelargonidin-*O*-glucuronide (6.74) ↑ pelargonidin-3-*O*-glucoside (4.66) ↑ cyanidin-3-*O*-glucoside (12.02) 20 g FDS: ↑ pelargonidin-*O*-glucuronide (16.25) ↑ pelargonidin-3-*O*-glucoside (3.06) ↑ cyanidin-3-*O*-glucoside (7.12) 40 g FDS: ↑ pelargonidin-*O*-glucuronide (9.74) ↑ cyanidin-3-*O*-glucoside (3.87)
Ruel et al., 2013 (Canada) [75]	Double-blinded, crossover (*n* = 35)	45 ± 10 years 35M	Overweight	500 mL reduced-calorie cranberry juice	Consumed daily for 4 weeks	Ox-LDL	∅
Zunino et al., 2012 (USA) [76]	Double-blinded, crossover (*n* = 20)	29 ± 7 years 7M 32 ± 11 years 13F	Overweight	80 g frozen strawberries	Consumed as FDS powder in beverage two times per day for 7 weeks	TAS; ORAC; 8-isoprostane	∅
Edirisinghe et al., 2011 (USA) [77]	Single-blinded, crossover (*n* = 24)	51 ± 15 years 10M/14F	Overweight	34 g FDS powder	Consumed with challenge meal typical of Western eating patterns	Polyphenolic compounds	↑ pelargonidin sulfate ↑ pelargonidin-3-*O*-glucoside (NP)
McAnulty et al., 2005 (USA) [78]	Parallel (*n* = 20)	Control: 29 ± 4 years Intervention: 26 ± 3 years 20M	Overweight	250 mg blueberries	Consumed daily for 3 weeks	F_2_-isoprostanes; lipid hydroperoxides; FRAP	↓ lipid hydroperoxides (within group)
Paiva et al., 1998 (Brazil) [79]	Crossover (*n* = 8)	67 ± 1 years 8F	Overweight	240 g strawberries	Beverage consumed as breakfast before lunch and dinner	Lutein; zeaxanthin; cryptoxanthin; α-carotene; Β-carotene; lycopene	All within group: 11 h after consumption: ↓ lutein (NP) ↓ zeaxanthin (NP) ↓ cryptoxanthin (NP) ↓ lycopene (NP) 15 h from consumption: ↓ Β-carotene (NP)
Cao et al., 1998 (USA) [80]	Crossover (*n* = 8)	67 ± 1 years 8F	Overweight	240 g strawberries	Beverage consumed as breakfast before lunch and dinner	ORAC_PCA_; FRAP; TEAC; vitamin C^3^; urinary ORAC	4 h after consumption: ↑ ORAC_PCA_ (0.84) ↑ FRAP (0.61) ↑ vitamin C^3^ (0.71) 24 h after consumption: ↑ ORAC (0.36)

*All biomarkers are plasma or serum measurements unless otherwise stated in the table. All outcomes are reported as between group unless stated in the table as within group. The Cohen’s d is calculated at a 95% confidence interval.* * A third arm was included but did not intervene with berries. ↑: increase; ↓: decrease; ∅: no change; 8-OHdG: oxo-2′-deoxyguanosine; DPPH: 2,2-diphenyl-1-picryl-hydrazyl; F: female; FDS: freeze-dried strawberry; FRAP: ferric reducing ability of plasma; g: gram; GPx: glutathione peroxidase; GSH: glutathione; GSSG: glutathione disulfide; HNE: 4-hydroxynonenal-2-nonenal; h: hours; M: male; MDA: malondialdehyde; mg: milligram; mL: milliliter; NP: not provided due to omitted data; ORAC: oxygen radical absorbance capacity; ox-LDL: oxidized low density lipoprotein cholesterol; PCA: perchloric acid; SOD: superoxide dismutase; TAS: total antioxidant status; TEAC: trolox equivalent antioxidant capacity.

**Table 3:** Risk of bias of parallel studies.

Study	Randomization	Deviation from Intended Interventions	Missing Outcome Data	Measurement of Outcome	Selection of Reported Result	Overall Risk of Bias
Cho et al., 2020 [30]	Low	Low	Low	Low	Low	Low
Hsia et al., 2020 [31]	Low	Low	Low	Low	Low	Low
Chew et al., 2019 [65]	Low	Low	Low	Low	Low	Low
Usharani et al., 2019 [64]	Low	Low	Low	Low	Low	Low
Kim et al., 2018 [36]	Low	Low	Low	Low	Low	Low
Feresin et al., 2017 [37]	Low	Low	Low	Low	Low	Low
Johnson et al., 2017 [42]	Low	Low	Low	Low	Low	Low
Lee et al., 2017 [43]	Low	Low	Low	Low	Low	Low
Nair et al., 2017 [38]	Some concerns	Low	Low	Low	Low	Low
Paquette et al., 2017 [41]	Low	Low	Low	Low	Low	Low
Xie et al., 2017 [44]	Low	Low	Low	Low	Low	Low
Zanchet et al., 2017 [39]	Low	Low	Low	High	Low	Low
An et al., 2016 [45]	Low	Low	Low	Low	Low	Low
Basu et al., 2016 [46]	Low	Low	Low	Low	Low	Low
Eftekhari et al., 2016 [71]	Low	Low	Low	Low	Low	Low
Prymont-Przyminska et al., 2016 [70]	High	Some concerns	High	High	Low	High
Davinelli et al., 2015 [72]	Low	Low	Low	Low	Low	Low
Johnson et al., 2015 [48]	Low	Low	Low	Low	Low	Low
Khazaal et al., 2015 [73]	Some concerns	Some concerns	Low	Low	Low	Some concerns
Basu et al., 2014 [50]	Low	Low	Low	Low	Low	Low
McAnulty et al., 2014 [49]	High	High	Low	Low	Low	High
Soltani et al., 2014 [51]	Low	Low	Low	Low	Low	Low
Basu et al., 2011 [55]	Low	Low	Low	Low	Low	Low
Puupponen-Pimiä et al., 2013 [52]	Some concerns	Low	Low	Low	Some concerns	Some concerns
Simão et al., 2013 [54]	Some concerns	High	High	Low	Low	Some concerns
Basu et al., 2010 [56]	Low	Some concerns	Low	Low	Low	Some concerns
Broncel et al., 2010 [58]	High	High	Low	Some concerns	Some concerns	High
Karlesn et al., 2010 [57]	Low	Some concerns	Low	Some concerns	Low	Some concerns
Ruel et al., 2006 [63]	High	Some concerns	High	High	Low	High
McAnulty et al., 2005 [78]	Some concerns	Low	Low	Low	Low	Low

**Table 4:** Risk of bias of crossover studies.

Study	Randomization	Period and Carryover Effects	Deviation from Intended Interventions	Missing Outcome Data	Measurement of Outcome	Selection of Reported Result	Overall Risk of Bias
Basu et al., 2021 [27]	Low	Low	Low	Low	Low	Low	Low
Marin-Echeverri et al., 2021 [29]	Some concerns	Low	Low	Low	Low	Low	Low
Richter et al., 2021 [28]	Low	Low	Low	Low	Low	Low	Low
Quintero-Quiroz et al., 2019 [33]	Some concerns	Low	Low	High	High	Low	Some concerns
Xiao et al., 2019 [32]	Some concerns	High	High	High	Some concerns	High	High
Basu et al., 2018 [66]	Low	Low	Low	Low	Low	Low	Low
Espinosa-Moncada et al., 2018 [35]	Low	Low	Low	Low	Low	Low	Low
Marin-Echeverri et al., 2018 [34]	Some concerns	Some concerns	Low	High	Some concerns	High	High
Richter et al., 2017 [68]	Low	Low	Low	Low	Some concerns	Some concerns	Low
Stote et al., 2017 [40]	Low	Some concerns	High	Low	High	High	High
Zhong et al., 2017 [67]	Some concerns	Low	Low	Low	Low	Low	Low
Huang et al., 2016 [69]	Low	Some concerns	Low	Low	Low	Low	Some concerns
Park et al., 2016 [47]	Some concerns	Some concerns	Low	Low	Low	Low	Low
Banaszewski et al., 2013 [74]	Low	Low	Low	Low	Low	Low	Low
Riso et al., 2013 [53]	Some concerns	Low	Low	Low	Low	Low	Low
Ruel et al., 2013 [75]	Low	High	Low	Low	Low	High	High
Zunino et al., 2012 [76]	Low	High	Low	Low	Low	High	Some concerns
Edirisinghe et al., 2011 [77]	Low	Low	Low	Low	Low	Low	Low
Burton-Freeman et al., 2010 [59]	Low	Low	Low	Low	Low	Low	Low
Jenkins et al., 2008 [60]	Low	Some concerns	Some concerns	Low	Low	Low	Some concerns
Ruel et al., 2008 [61]	High	High	High	Low	Some concerns	High	High
Skoczyñska et al., 2007 [62]	High	Low	High	Low	Some concerns	High	High
Cao et al., 1998 [80]	Low	Low	Some concerns	Low	Low	Low	Some concerns
Paiva et al., 1998 [79]	Some concerns	Low	Some concerns	Low	High	Low	Some concerns

**Table 5:** Summary findings for applications in clinical practice and public health recommendations.

Recommended Berry Dosage	Oxidative Stress Benefits	Antioxidant Benefits	Benefits to Cardiometabolic Risk Factors
100 mg aronia extract	Reduced lipid peroxidation byproducts [58]	Increased enzymatic activity [58]	Reduced blood pressure [58] Increased high-density lipoprotein cholesterol [58]
22 g blueberries (freeze-dried)	Reduced cellular oxidative stress [42] Reduced lipid peroxidation byproducts [48,56]	Increased enzymatic activity [48]	Reduced blood pressure [48,56]
250 mL chokeberry juice	None reported	Increased antioxidant concentration [62]	Reduced triglycerides [58] Reduced serum glucose [62]
240 mL cranberry juice	Reduced lipid peroxidation byproducts [55]	Increased antioxidant capacity [55]	Reduced waist circumference [63] Increased high-density lipoprotein cholesterol [63]
14 g goji berry	Reduced lipid peroxidation byproducts [39]	Increased enzymatic activity [39] Increased antioxidant concentration [39] Increased antioxidant capacity [39]	Reduced waist circumference [39]
500–1000 mg Indian gooseberry	Reduced lipid peroxidation byproducts [64]	Increased enzymatic activity [64]	Reduced triglycerides [64]
500 mg raspberry ketones	None reported	Increased antioxidant concentration [73]	Reduced waist circumference [73]
10–25 g strawberries (freeze-dried)	Reduced lipid peroxidation byproducts [50]	Increased enzymatic activity [27,46] Increased antioxidant capacity [46] Increased antioxidant concentration [74]	None reported
454 g strawberries	Reduced lipid peroxidation byproducts [60]	None reported	None reported
500 mg whortleberry (dried)	Reduced lipid peroxidation byproducts [51]	None reported	Reduced triglycerides [51]
